# Scalable high-precision tuning of photonic resonators by resonant cavity-enhanced photoelectrochemical etching

**DOI:** 10.1038/ncomms14267

**Published:** 2017-01-24

**Authors:** Eduardo Gil-Santos, Christopher Baker, Aristide Lemaître, Carmen Gomez, Giuseppe Leo, Ivan Favero

**Affiliations:** 1 Matériaux et Phénomènes Quantiques, Université Paris Diderot, CNRS UMR 7162, Sorbonne Paris-Cité, 10 rue Alice Domon et Léonie Duquet, Paris 75013, France; 2 Laboratoire de Photonique et de Nanostructures, Route de Nozay, CNRS, Marcoussis 91460, France

## Abstract

Photonic lattices of mutually interacting indistinguishable cavities represent a cornerstone of collective phenomena in optics and could become important in advanced sensing or communication devices. The disorder induced by fabrication technologies has so far hindered the development of such resonant cavity architectures, while post-fabrication tuning methods have been limited by complexity and poor scalability. Here we present a new simple and scalable tuning method for ensembles of microphotonic and nanophotonic resonators, which enables their permanent collective spectral alignment. The method introduces an approach of cavity-enhanced photoelectrochemical etching in a fluid, a resonant process triggered by sub-bandgap light that allows for high selectivity and precision. The technique is presented on a gallium arsenide nanophotonic platform and illustrated by finely tuning one, two and up to five resonators. It opens the way to applications requiring large networks of identical resonators and their spectral referencing to external etalons.

## Abstract (web summary)

Aligning the resonances of sets of optical cavities is necessary for advanced photonics and sensing applications. Here, the authors introduce resonant photoelectrochemical etching as a method to collectively and permanently tune the resonant wavelengths of ensembles of resonators on a photonic chip.

The advent of microfabrication and nanofabrication techniques has had a profound impact on the control of light–matter interactions, be it in semiconductor, metallic or insulating materials. It is today possible to tailor the optical properties of emitters by engineering their coupling to solid-state resonant cavities, waveguides or antennas. Collective photonic architectures based on microscale and nanoscale resonators serve in many contexts, such as information processing^1^,^2^,^3^, sensing^4^, metamaterials^5^, polaritonics^6^, nonlinear optics^7^, optomechanics^8^ and plasmonics^9^. Lattices of resonant cavities are also becoming a test bed for the physics of strongly correlated optical systems^10^, with a recent focus on synchronization^11^, phase-transition^12^ and topological phenomena^13^. However, in all these recent applications, there is still a gap between ideas and experimental realizations owing to insufficient deterministic control in the resonant optical wavelength of single and multiple resonators, which precludes many of the above concepts from becoming reality. Technologically speaking, conventional nanofabrication tools such as electron-beam lithography or ion milling and etching typically allow for a nanometre-scale precision in the dimensions of the finished device at best. For microscale and nanoscale cavities, such an imprecision translates into a sizeable uncertainty in the resonant optical wavelength. As an example, let us consider a whispering gallery mode (WGM) disk resonator of nominal radius *R*=1 μm, fabricated out of a strongly refractive semiconductor-like gallium arsenide (GaAs), and resonating at a wavelength *λ*_WGM_≈1 μm (refs 14, 15). As *δλ*_WGM_/*λ*_WGM_=*δR*/*R* (ref. 14), with *δR* the radius imprecision, the near-infrared optical modes of such a disk, once fabricated, also face an imprecision *δλ*_WGM_ of a few nanometres in their resonance. For two resonators to share a common resonance frequency, a precision *δR*<*R*/*Q* is required, with *Q* the resonator quality factor. Even with a modest *Q*=10^4^, this means a resonator size control at the level of the Angstrom, the level of a single atom. In consequence, two nominally identical cavities, once fabricated, always resonate at distinct wavelengths, precluding collective spectral alignment or resonant interaction with targeted references. This disorder is a major obstacle for the future of nanophotonics. It needs to be overcome, ideally with a technique simple enough to spread from basic research experiments to industrial settings, where complex photonic architectures will demand reasonable technological complexity in the handling.

Several techniques have been proposed to partially address these issues. Gas adsorption and surface desorption and oxidation allowed, for example, tuning individual miniature optical resonators^16^,^17^,^18^,^19^,^20^,^21^. Thermal^11^,^22^, electrical^23^,^24^ and mechanical^25^ tuning of optical micro-resonators was also demonstrated but requires continuous energy consumption. These latter approaches produce non-permanent effects and are difficult to scale up. The tuning of photonic structures was also obtained by functionalization with a photochromic film^26^, a laser-addressable polyelectrolyte^27^ or a photosensitive layer^28^. However, these techniques complicate the handling of multiple cavities and tend to degrade optical and mechanical properties by surface modification. Ultraviolet light at or above the bandgap was used to photo-etch gallium nitride devices^29^,^30^,^31^ and trim silicon optical structures^32^, however, with limited precision. Fluidic tuning was achieved by water infiltration and evaporation at the level of individual photonic cavities but with little permanence^33^. Importantly, all these methods are poorly scalable.

Here we present a new method to achieve spectral tuning of microphotonic and nanophotonic resonant cavities, which solves all of the above shortcomings. The method introduces the concept of resonant cavity-enhanced photoelectrochemical (PEC) etching, whereby the dielectric material forming the optical cavity is etched only in the presence of light resonating in the cavity and when immersed in a fluid. In contrast to conventional PEC configurations that use light above the bandgap, this new technique operates in the transparency region of the material. This latter fact enables taking advantage of the high-*Q* resonances of the cavities to be tuned. When a cavity is driven at resonance by a laser, a strong internal light enhancement takes place and triggers an enhanced etching selectively within the cavity, leading to its tuning. The technique can operate with a single laser, produces permanent results with no optical performance degradation and is automatically scalable to multiple resonators. Being adjustable by light intensity, it allows both fine-tuning of a resonator's wavelength at a picometre level and coarse-tuning over tens of nanometres, both within a few minutes of time. The method is both site-specific, in that it resonantly addresses each individual resonator of an inhomogeneous ensemble, and collective, because it does not require individual identification of each resonator to achieve spectral alignment of the ensemble. We introduce the method on a GaAs photonic platform, a material that allows resonant PEC etching in water. To establish the performance of the method, we investigate first the tuning of an individual resonator and then demonstrate the collective tuning of an ensemble of five semiconductor photonic cavities immersed in liquid water. We finally explore a variation of the technique in a simple humid atmosphere, showing that versatility in application of PEC methods is obtainable with alleviated fluidic constraints.

## Results

### Resonant PEC etching of GaAs disk resonators

**Figure 1:**
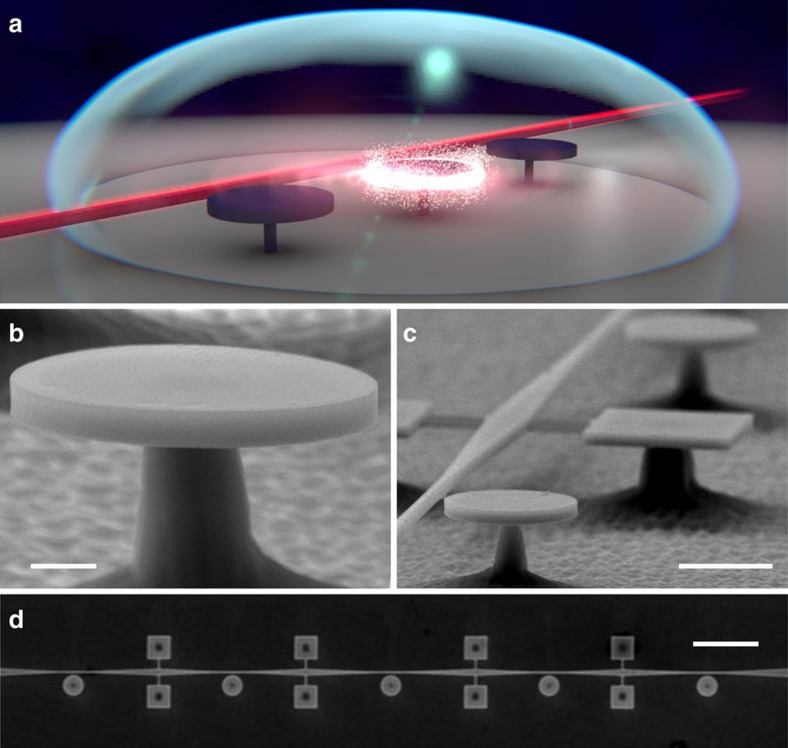
Resonant PEC tuning of GaAs disk resonators on a chip. (**a**) Illustration of the resonant PEC etching of a disk resonator coupled to a linear optical waveguide. Laser light is in red while the shining zone represents a whispering gallery mode where electromagnetic energy localizes and triggers the cavity etching. The disk belongs to a set of three resonators immersed in a liquid droplet. (**b**) Side-view electron micrograph of a single GaAs disk positioned on top of a central AlGaAs pedestal. Scale bar 500 nm. (**c**) Set of two disks positioned along a common GaAs suspended optical waveguide. The waveguide's tapered parts lie close to the disks while square-shape anchors support its widest part. Scale bar 2 μm. (**d**) Top-view electron micrograph of five disk resonators in series along a waveguide held by eight square anchors. Scale bar 10 μm.

**Figure 2:**
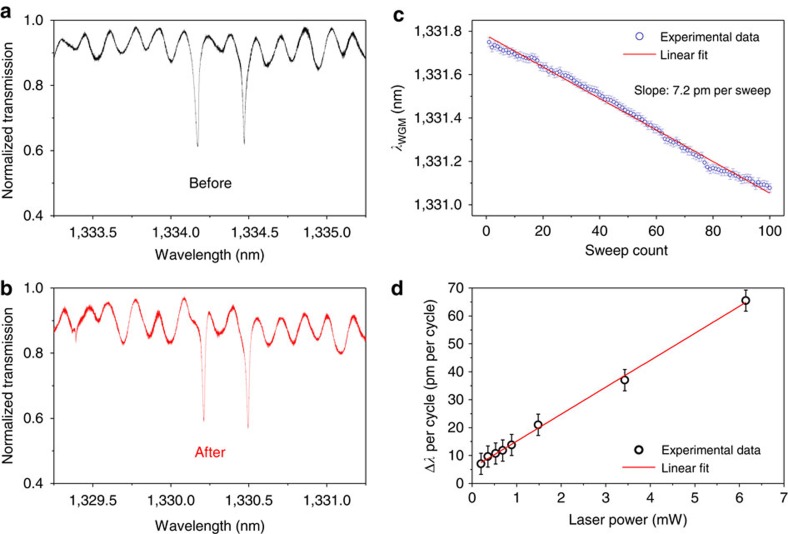
Resonant PEC tuning of a single GaAs WGM disk resonator. (**a**) Optical spectrum before resonant PEC etching, with a doublet associated to two normal modes of the disk, consisting of a mixture of clockwise and counterclockwise WGMs^15^. (**b**) Optical spectrum after 4 nm of resonant PEC tuning. The low-contrast oscillations are residual interferences produced by reflections at the waveguide input and output facets. They can be mitigated by proper guide design. (**c**) Wavelength tuning of the WGM of a GaAs disk resonator immersed in water by subsequent cycles of resonant PEC etching at low optical power, reaching picometre precision. This translates into an etch speed of 0.5–1 nm s^−1^ for a continuous injected power of 1 μW in the cavity WGM. (**d**) Wavelength tuning per cycle (see text) as a function of the laser optical output power, which is proportional to the optical power guided in the coupling waveguide. Each point has been obtained by averaging over 100 cycles, the shown error bar is the related s.d.

The results reported in the following are obtained with GaAs disk resonators, whose typical dimensions are of 320 nm in thickness and 1 μm in radius^34^,^35^,^36^. These resonators can support WGMs of mode volume <1 μm^3^ and represent archetypes of microphotonic/nanophotonic cavities for the purposes discussed here. An illustration of the resonant PEC etching method applied to GaAs disks is given in Fig. 1a, where one individual resonator is selectively etched and tuned by laser light, while the other two are out of resonance and remain in essence unaffected (see Supplementary Note 3 for more details). Our GaAs disk resonators are isolated from the substrate by an aluminum gallium arsenide pedestal, as shown in Fig. 1b. They are fabricated out of a GaAs (320 nm)/Al_0.8_Ga_0.2_As (1,800 nm)/GaAs epitaxial wafer by electron beam lithography, non-selective inductively-coupled plasma reactive ion etching and selective under-etching in a dilute hydrofluoric acid solution. GaAs waveguides integrating a taper with nanoscale transverse dimensions allow evanescent optical coupling of light into the disk's WGMs^37^ and are suspended in the resonator's vicinity (see Fig. 1c,d). Owing to the large refractive index of GaAs, the chip can be immersed in a transparent liquid while preserving the confinement and guiding of light in the resonators and waveguides. The presence of a microlitre droplet of liquid covering many resonators still allows optical evanescent coupling disk/waveguide experiments to be performed *in situ*^38^,^39^. This is the first approach that we employ to tune our disk resonators by resonant cavity-enhanced PEC (see Supplementary Fig. 1). The disk WGMs appear as resonant dips in the waveguide's optical transmission spectrum (see Fig. 2a). In Fig. 2a, the linewidth of both visible resonances is 32 pm in ambient conditions before liquid immersion, corresponding to a loaded WGM quality factor *Q* of 41,000. At resonance, the light-assisted PEC etching of the cavity is resonantly favoured over the etching of the waveguide (see Supplementary Note 3). The etching reduces the size of the disk resonator and blue shifts its optical resonances. This shift can be continuously controlled and monitored in time in the liquid by continuously sweeping the laser wavelength over the resonance. A real-time movie of such continuous monitoring is shown in Supplementary Movie 1. Once the desired amount of etching is reached, the laser is switched off and the sample dried. The dry resonator's optical spectrum, whose overall structure is preserved, reveals a permanent blue shift of 4 nm (see Fig. 2b). The linewidth of the two resonances is now of 19 and 25 pm, respectively, with an almost unaltered contrast. The loaded *Q*s rise to 70,000 and 52,000, which represents a marked reduction of optical losses with respect to the situation before etching. We carried out similar tests on many resonators, showing that the optical *Q* may be improved by the resonant cavity-enhanced PEC tuning and is at least never degraded. Let us now discuss the mechanisms at play in our tuning technique that are responsible for these advantageous features.

### Mechanism and performance of resonant PEC etching

In standard (non-resonant) PEC etching, one optically pumps, above the bandgap, a semiconductor immersed in an electrically conductive liquid. Ultraviolet light is generally used to generate carriers leading to the formation of ionic species of the semiconductor. The latter are dissolved in the presence of ions provided by the electrolyte liquid^40^,^41^,^42^, which can be water in the case of GaAs. The favoured chemical reaction for intrinsic GaAs reads GaAs→GaAs^3+^+As^3+^+6e^−^, whose products form oxides with the hydroxide ions of water and are then removed^41^. In the present work, in contrast, we achieve resonant PEC etching in liquid with light of frequency in the transparency region of the material. The reason is that high-*Q* GaAs disk resonators, just as all equivalent high-*Q* photonic cavities, can absorb a (small) fraction of the resonating light they store at resonance. In GaAs disks, a residual linear absorption is, for example, probably caused by mid-gap states localized at the resonator's surface^39^. In consequence, the ionic species required for triggering the PEC etching can be generated while optically pumping below the material bandgap, at the resonance of a WGM. This resonant cavity-enhanced nature of our PEC tuning brings in key advantages. For example, it leads to high spatial selectivity by favouring the etching precisely within the optical mode of the selected resonator. This spatial localization of the process has several beneficial consequences: in the case of a WGM localized at the periphery of a disk, it may be responsible for the *Q* improvement observed in our experiments, by smoothing out geometric irregularities of the disk contour.

Another benefit is that a small flux of light can be used to etch each resonator, allowing for a high level of control in the amount of tuning, and hence a high tuning precision. This is exemplified in Fig. 2c, which demonstrates the spectral accuracy of the resonant PEC tuning technique by employing a series of elementary fine-tuning cycles. Each cycle consists of rapidly sweeping the wavelength of the laser back and forth over the WGM resonance, which allows for both acquiring an optical spectrum and step tuning the resonating wavelength. Figure 2c shows a resonance wavelength measured on a GaAs disk WGM, as a function of the number of applied cycles. The evolution is linear in the number of cycles, with a blue shift of 7.2 pm per cycle. For the disk considered above, and translated into an effective change of radius, this represents a size control with a precision of 8 pm per cycle, which is well below the material's interatomic distance. More precisely, each of these sweep cycles reduces the disk's size by less than a thirtieth of an atomic monolayer (280 pm). In contrast, the simple process of immersing the chip in an acidic solution to remove the native surface oxide would shift the same WGM optical resonance by approximately 2 nm (refs 39, 43). The sweep-cycle tuning procedure outlined here is >250 times more precise. By calculating the effective etching duration per cycle, we find an etching speed of 0.5–1 nm of radius change per second for 1 μW of continuous optical power injected into the cavity WGM. By increasing the laser power, one can adjust the amount of tuning per cycle in a linear manner, as shown in Fig. 2d. Note that the intercept of the linear fit does not cross the origin, which is mainly ascribed to a very slow non-selective GaAs etching in water (see discussion below and Supplementary Note 3). By choosing the light intensity and the number of cycles, the resonance wavelength can be tuned between a few picometres and several tens of nanometres, with permanent results, and within just a few minutes. It is worth noting that the sweep-cycle mode is not the only procedure that can be used for tuning. Another possibility is to have the laser wavelength continuously following the optical resonance as it is tuned. At low optical power, our experiments indicate that this latter procedure leads to a tuning precision even better than the picometre, currently beyond the spectral accuracy at our disposal in the laboratory. This great spectral versatility of the technique in terms of tunability and precision also comes with other benefits when ensembles of resonating cavities are handled, which we discuss now in more detail.

### Resonant PEC tuning of ensembles of resonators

**Figure 3:**
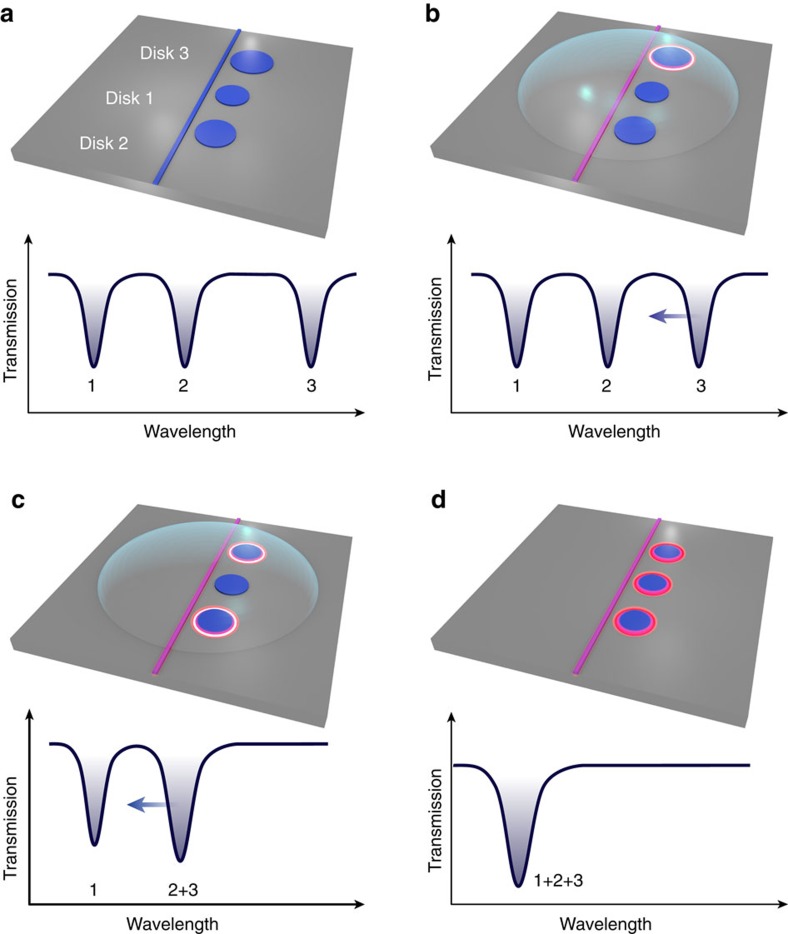
Scalability of the resonant cavity-enhanced PEC tuning explained for three cavities in series. (**a**) An optical bus waveguide couples in series to three disk cavities having distinct radius, hence distinct optical resonant wavelength. The optical spectrum consists of three resonances. (**b**) The cavities are immersed in a PEC liquid and the laser wavelength is set to the largest resonant wavelength. This triggers resonant PEC etching in the disk of largest radius (Disk 3), progressively reducing its radius and blue shifting its resonance (blue arrow). (**c**) When Disk 3 becomes resonant with Disk 2, the resonant cavity-enhanced PEC etching becomes concomitant in these two disks and displaces their optical resonance towards that of the smallest disk of the set (Disk 1). (**d**) At a certain point, Disks 2 and 3 become resonant with Disk 1, providing us with a set of three spectrally aligned cavities.

Indeed one crucial aspect of the method is its natural scalability, which is obtained with a single laser and with relatively modest efforts. The scalability is a consequence of the resonant nature of our cavity-enhanced tuning process and is easily understood in the case of three resonators (see Fig. 3). Let us imagine three distinct disk resonators (1–3) of increasing radius, hence increasing resonant wavelength (Fig. 3a). Let us immerse them in a PEC-enabling fluid and set the laser in resonance with Disk 3 (Fig. 3b). The cavity-enhanced PEC etching starts preferentially in Disk 3 and reduces its radius. As its resonant wavelength is, in consequence, blue-shifted, we continuously and slowly sweep the laser towards smaller wavelengths in order to track the resonance and sustain the etching, until Disk 3 adopts the resonant wavelength of Disk 2. From that moment on, Disks 2 and 3 are resonantly etched together by the laser and are progressively tuned to lower wavelengths (Fig. 3c). When their common resonant wavelength reaches that of Disk 1, the three disk resonators are spectrally aligned (Fig. 3d). The principles of this scalable tuning can be extended to a larger number of resonators, using a single laser and a single laser wavelength sweep, and they enable collective spectral alignment without having to individually identify each resonator of the set (that is, the tuning procedure remains the same irrespective of the order of disks 1,2,…,*N* along the waveguide). This is in strong contrast with other tuning methods investigated so far. While the case of three resonators is used for illustrative purposes in Fig. 3 (see also Supplementary Note 2 and Supplementary Figs 3 and 4), below we experimentally demonstrate the scalability of the process with five cavities.

**Figure 4:**
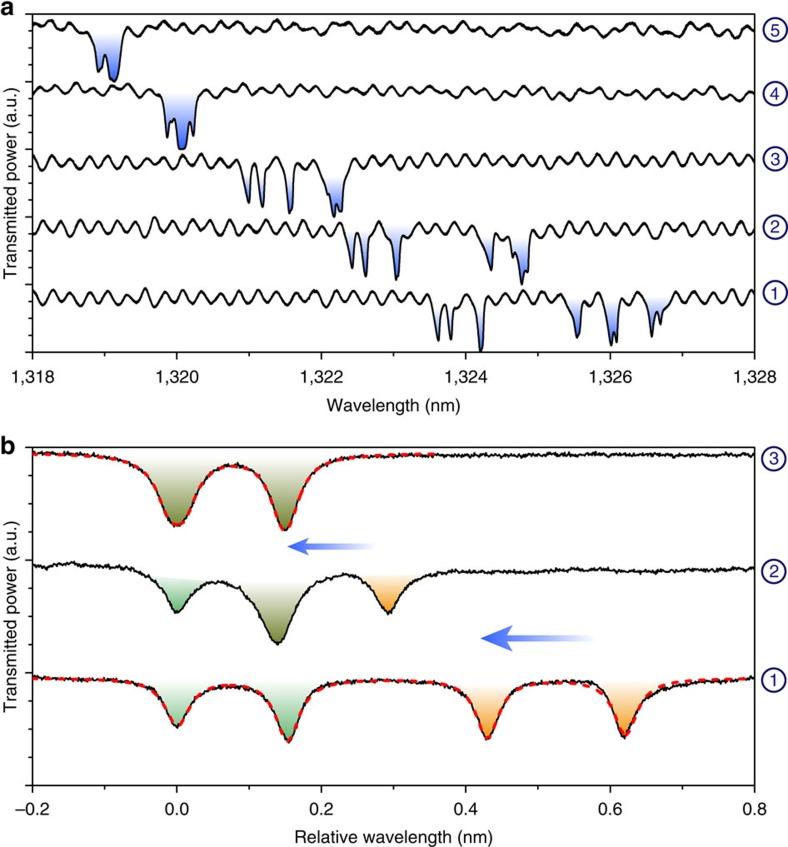
Collective resonant PEC tuning of photonic resonators. (**a**) Five optical spectra corresponding to step-by-step spectral alignment of five WGM resonators in liquid water. In the first spectrum, the five resonators (1–5) appear in order of their resonant wavelength (*λ*_1_<…<*λ*_5_). In addition to the spectral merging of the five resonators, a residual non-selective etching is also present and discussed in full in Supplementary Note 3 and in Supplementary Fig. 5. (**b**) Spectral tuning of two WGM resonators, performed directly in humid air without liquid. In these three latter spectra relative wavelengths are displayed, with the origin being set by the smallest wavelength of the group of resonances. Initially (step 1), the two WGM doublets are disjoint before they begin to merge (step 2), resulting in three transmission dips whose overlap is coloured green. Finally (step 3), both WGM doublets overlap completely. As the resonant cavity-enhanced PEC tuning in humid air is a slow process, we removed here the contribution of slow temperature variations in the laboratory, which typically red-shift all wavelengths by 200 pm for 1 °C of temperature variation (see Supplementary Fig. 6 for extra raw data). The blue arrows indicate the spectral tuning direction. The red dashed line is a model (see text).

Figure 4a shows a series of five optical spectra acquired on a set of five distinct GaAs disks placed along a common optical coupling waveguide in the configuration of Fig. 1d and immersed in liquid water. The first spectrum (step 1) contains five groups of resonances, each group being associated to a distinct disk. Three groups display a clearly visible doublet structure, whereas this structure is not clearly resolved in the two others. Even though the five disks are nominally the same, they optically resonate at distinct wavelengths (*λ*_1_<…<*λ*_5_), because of imperfections in nanofabrication. The largest disk (Disk 5) resonates at the largest wavelength *λ*_5_, around 1,326.8 nm. By setting the laser to this wavelength, we trigger the resonant PEC etching in Disk 5 and blue shift its resonances, until it spectrally aligns with Disk 4 (step 2). From that stage on, the laser etches Disks 5 and 4 concomitantly and spectrally drags their aligned resonances towards those of Disk 3. At step number 3, the resonances of Disks 5, 4 and 3 merge and the laser starts etching the three disks together, towards Disk 2. The procedure of tuning and spectrum acquisition is repeated this way, progressively bringing all the disks to spectrally align. At step 5, the five disks have their resonances spectrally overlapped, and the laser can be switched off. On top of the spectral aligment, we observe that a non-selective tuning takes place along the procedure, generating an additional blue shift of the set of resonances. The origin of this non-selective tuning is discussed in detail in Supplementary Note 3 and Supplementary Fig. 5. It can be mitigated if required for a specific application. The final result of the tuning procedure is a set of five spectrally aligned cavities. It is important to note that, while five steps are discussed in Fig. 4a in order to illustrate the process, the PEC spectral alignment can also be performed by a single slow continuous laser sweep starting from the red-detuned side of the resonances. This continuous collective tuning mode is illustrated by a simulation in Supplementary Movie 2.

Although PEC tuning in a liquid, as demonstrated above, may suffice in a number of applications, the constraints associated with liquid immersion may pose problems for some cavity geometries and for some photonic materials. For this reason, in Fig. 4b, we explore a variation of the method in a gaseous environment that circumvents liquid immersion. With GaAs, the humidity of ambient air is sufficient to trigger a very slow PEC tuning process under resonant optical pumping of the cavity. At equal pumping power, we have evaluated that in this latter case the tuning speed is six orders of magnitude slower than in water. Intermediate speeds can be obtained by controlling the water content of the surrounding atmosphere. Figure 4b shows the spectral tuning of two distinct GaAs disk resonators under ambient humidity conditions in the configuration of Fig. 1c. At step 1, we observe two groups of doublets, each group being associated with a given disk. At the intermediate step 2, these resonances partially overlap. At the final step (step 3), the two doublets are perfectly overlapped. The two disks are now, once and for all, resonating at the same optical wavelength down to a precision better than 10 pm. The dashed lines are a fit provided by a simple model of cascaded resonators coupled to a common optical waveguide and support that the tuning procedure follows the ordering principles introduced above. With the slow speed of tuning reached in these conditions, the tuning procedure becomes time consuming (10 h of experiments here), but it allows a level of spectral control that is even superior to the picometre precision reached in the liquid in Fig. 2. For each specific application, a compromise between precision and speed will need to be made by adjusting the density of ionic species in the fluid environment (liquid or gas) used to sustain the PEC processes.

## Discussion

The collective tuning method can be naturally scaled to more than five resonators, again because spectral alignment does not require singling out each resonator. The precision and selectivity of the method can be improved as well beyond what we report here. As detailed in Supplementary Note 1 and Supplementary Fig. 2, the tuning precision of a few picometres reached in water in the sweep-cycle mode is, in our case, set by the absolute spectral inaccuracy of our laser and could be improved by improved laser referencing. The selectivity is limited by residual non-selective tuning that could be mitigated as well (see Supplementary Note 3). As we observed resonant PEC etching of GaAs disks in ammonia and isopropanol as well (not shown), we know that other ionic liquids can be employed to gain even better control on the technique. As demonstrated by our tuning experiments in humid air, we also understand that controlled ionic gas atmospheres may enable many variations of the method. On top of this, PEC processes occur in many dielectric semiconductors, and the method of resonant cavity-enhanced PEC tuning introduced here with GaAs is in principle transposable to other material platforms. For the case of silicon, the water-rich fluidic environment used in this work should be replaced by a fluoride-based liquid or vapour to adapt the technique^44^. Specific optimization will be needed for each semiconductor showing PEC capability, such as silicon, gallium-nitride or antimonide, zinc-sulfide or selenide, to name a few. The method is, in essence, also adaptable to different photonic cavity architectures, for example, those based on photonic crystals, rings or slots. Overall, because the resonant PEC tuning technique is site-specific, precise, naturally collective and gives permanent effects, it may become a powerful tool for nanoscale photonic devices and motivate extensions to many materials and structures. The field of nanophotonics will require advances of the kind to hold all its promises.

### Data availability

The authors declare that the data supporting the findings of this study are available within the paper and its Supplementary Information files.

## Additional information

**How to cite this article:** Gil-Santos, E. *et al*. Scalable high-precision tuning of photonic resonators by resonant cavity-enhanced photoelectrochemical etching. *Nat. Commun.* **8**:14267 doi: 10.1038/ncomms14267 (2017).

**Publisher's note**: Springer Nature remains neutral with regard to jurisdictional claims in published maps and institutional affiliations.

## Supplementary Material

Supplementary Information Supplementary Figures, Supplementary Notes and Supplementary References

Supplementary Movie 1 Real-time video of the experimental resonant PEC tuning of the whispering gallery doublet resonance of a single GaAs disk resonator.

Supplementary Movie 2 Video illustration of spectral self-alignment of three distinct photonic resonators by resonant PEC tuning.
