# Author Correction: Scalable high-precision tuning of photonic resonators by resonant cavity-enhanced photoelectrochemical etching

**DOI:** 10.1038/s41467-018-05788-z

**Published:** 2018-08-28

**Authors:** Eduardo Gil-Santos, Christopher Baker, Aristide Lemaître, Sara Ducci, Carmen Gomez, Giuseppe Leo, Ivan Favero

**Affiliations:** 1 Matériaux et Phénomènes Quantiques, Université Paris Diderot, CNRS UMR 7162, Sorbonne Paris-Cité, 10 rue Alice Domon et Léonie Duquet, 75013 Paris, France; 2 Laboratoire de Photonique et de Nanostructures, Route de Nozay, CNRS, 91460 Marcoussis, France

Correction to: *Nature Communications* **8**:14267; 10.1038/ncomms14267; published online 24 Jan 2017

The original version of this Article omitted the fourth author, Sara Ducci from Matériaux et Phénomènes Quantiques, Université Paris Diderot, CNRS UMR 7162, Sorbonne Paris-Cité, 10 rue Alice Domon et Léonie Duquet, Paris 75013, France. This mistake has been corrected in both the HTML and PDF versions of the Article.

