# Supplementary material for: Scalable high-precision tuning of photonic resonators by resonant cavity-enhanced photoelectrochemical etching
Source: Nat Commun. 2017 Jan 24;8:14267. doi: 10.1038/ncomms14267 (PMC5286200; doi:10.1038/ncomms14267)
Supplement: Supplementary Information — Supplementary Figures, Supplementary Notes and Supplementary References [file ncomms14267-s1.pdf]

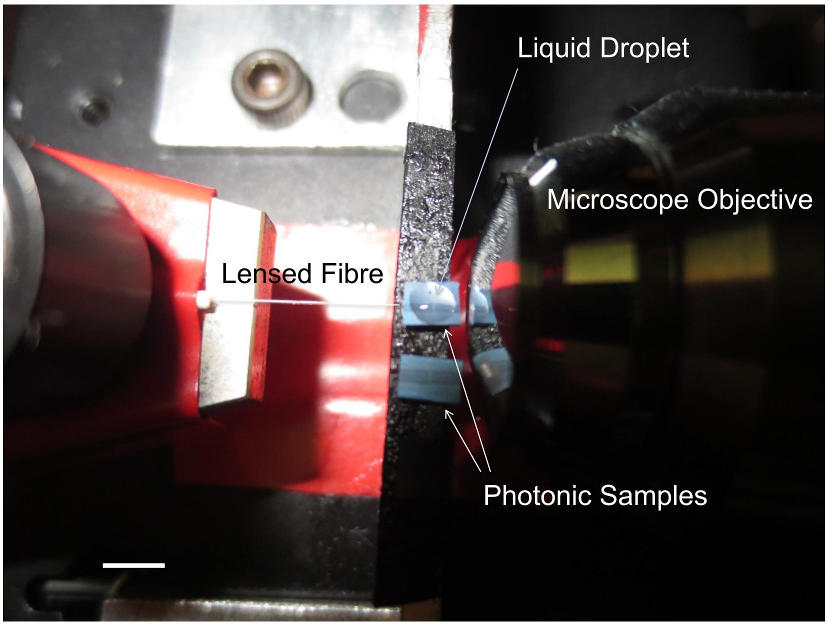

Supplementary Figure 1. **Resonant photoelectrochemical (PEC) etching set-up.** Binocular top-view of the set-up employed for the resonant PEC tuning of GaAs disk resonators. Two semiconductor photonic samples are placed on a sample holder (in black). A liquid droplet is formed on the upper sample. This latter sample is sandwiched between an optical micro-lensed fibre for light injection into the sample's waveguides (left) and a microscope objective for light collection at the waveguide's output (right). The lensed fibre is connected to a tunable external cavity diode laser (Tunics model, continuous-wave, mode-hope-free, line width of 400 kHz) and the light collected by the objective focused back on a p-i-n photodetector. The sample holder, lensed fibre and objective are mounted on *xyz* micro-positioning stages for alignment. Scale bar 2 mm.

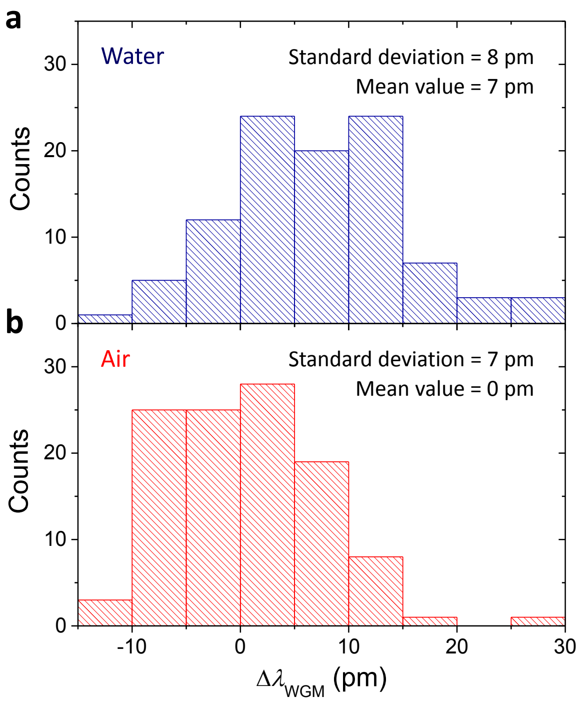

Supplementary Figure 2. **Laser and tuning precision.** (a) Histogram of the measured wavelength shift per cycle in water, built from a series of one hundred cycles of resonant PEC tuning in the sweep-cycle mode. (b) The same histogram is obtained from measurements performed in air, where PEC etching occurs with six orders of magnitude lower intensity.

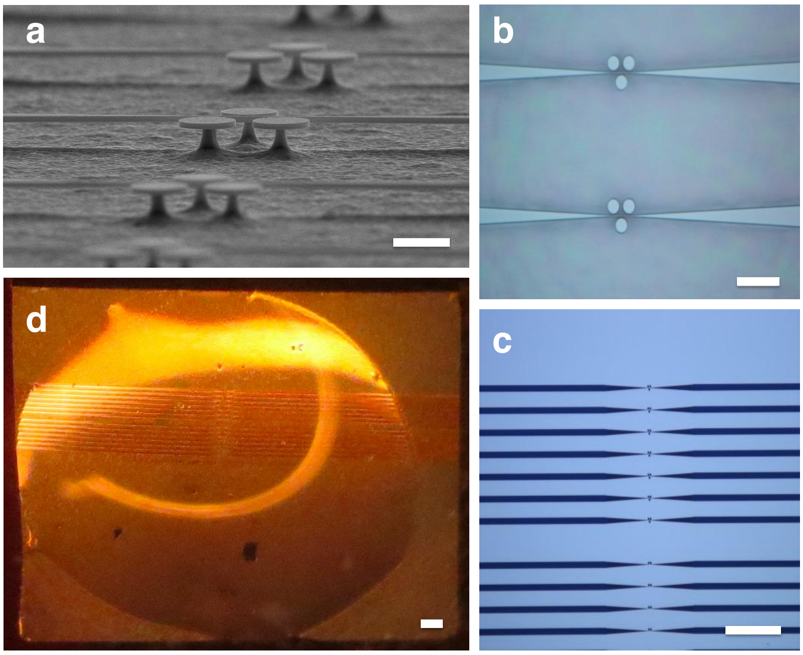

Supplementary Figure 3. **Three GaAs disk resonators on a chip for resonant PEC tuning in liquid.** (a) Side-view electron micrograph of sets of 3 disks positioned on both sides of a common GaAs suspended optical-coupling waveguide. Scale bar 3 µm. (b) Top-view optical micrograph of similar sets, with the tapering of the waveguide made visible. Scale bar 10 µm. (c) Top-view optical micrograph of the waveguides. Scale bar 100 µm. (d) Top-view optical micrograph of the chip immersed in a liquid droplet. Scale bar 100 µm.

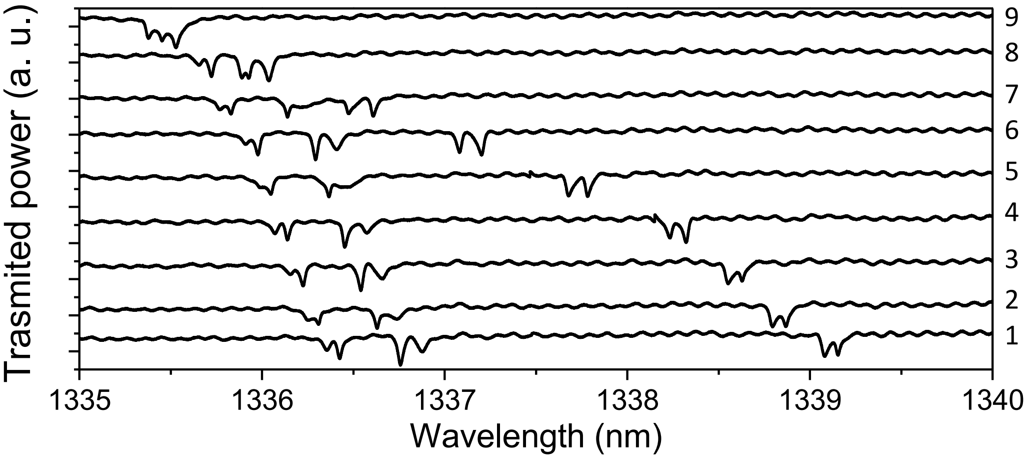

Supplementary Figure 4. **Collective tuning of three photonic resonators by resonant PEC etching in liquid water.** Optical spectra corresponding to step-by-step spectral alignment of three WGM resonators in the configuration shown in Supplementary Fig. 3. Each doublet corresponds to one disk to start with. The label on the right axis corresponds to the step number.

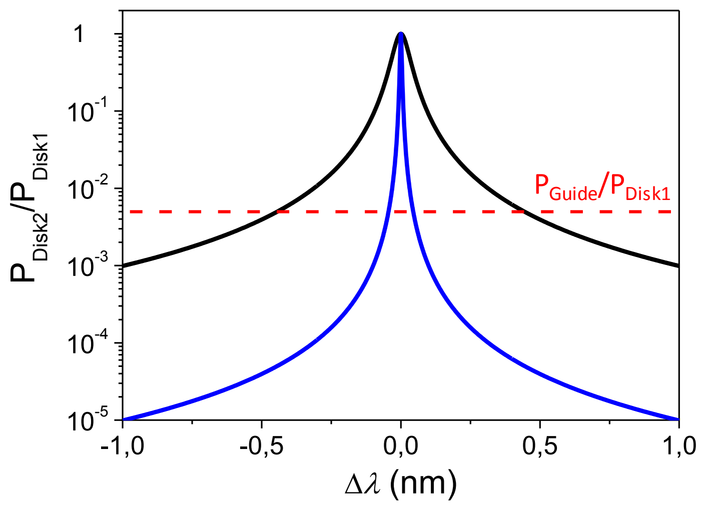

Supplementary Figure 5. **Relative optical powers circulating in two disks coupled to the same waveguide.** The WGM of Disk1 is resonantly pumped by the laser, the WGMs of both disks are detuned by $\Delta\lambda$, and the disk/waveguide evanescent coupling conditions are the same for both disks. The loaded Q is 22,000 (blue) or 220,000 (black), with a resonance contrast of 0.7.

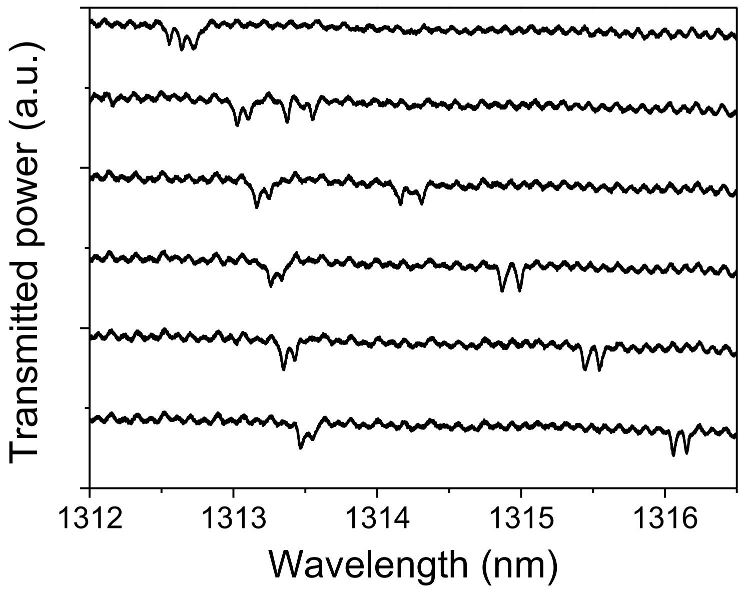

Supplementary Figure 6. **Resonant PEC tuning of two GaAs disk resonators in water in the sweep-cycle mode.** The duration of each cycle, between two spectrum acquisitions, is controlled to be reproducibly 2 minutes.

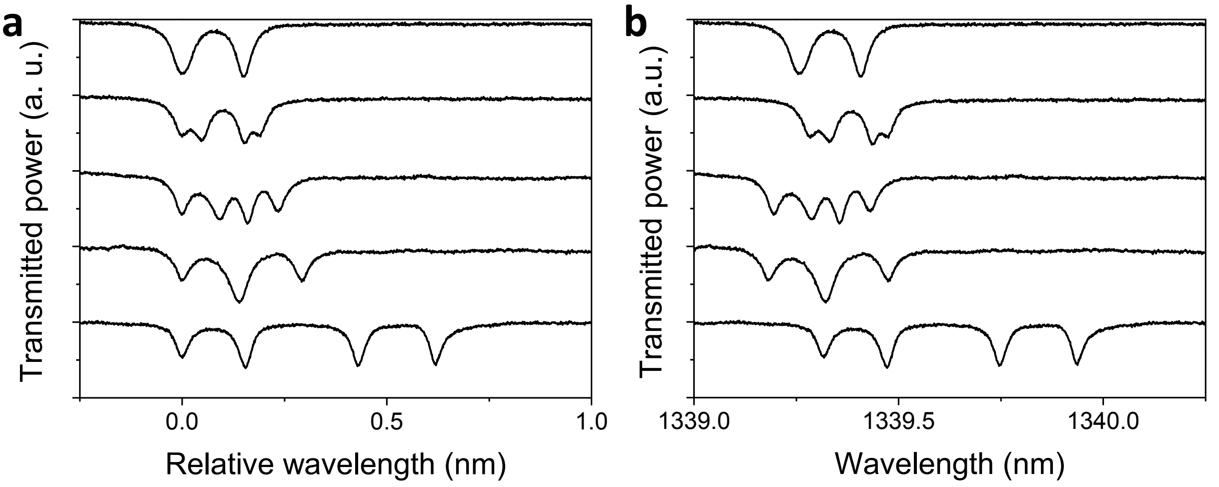

Supplementary Figure 7. **Intermediate steps of the resonant PEC tuning of two cavities in humid air.** (a) Resonant PEC tuning of two cavities in humid air, where the relative wavelength is shown in order compensate for thermal spectral wandering of the resonances. (b) The same tuning series, with the bare wavelength shown. This illustrates the spectral wandering taking place in this long (several hours) tuning procedure, as a consequence of temperature variations in the laboratory.

Supplementary Note 1: Laser imprecision and tuning

The results shown in the main text indicate that a wavelength shift of 7.2 pm per cycle is reached in the resonant PEC tuning process, when subsequent tuning cycles are used at low laser power (sweep-cycle mode). This wavelength shift per cycle must be understood as a mean value, as each point of Fig. 3(d) is obtained by averaging over 100 cycles. Here we show a statistical analysis of the measured per-cycle wavelength shift, to understand the origin of the residual imprecision of our tuning method. Supplementary Fig. 2(a) shows a histogram of the shift in a similar low optical power regime, derived from 100 measured cycles performed in water. This histogram shows a mean value of 7 pm for the (negative) wavelength shift. The standard deviation of the measurement amounts to 8 pm, which sets the experimental precision of our sweep-cycle tuning mode. Supplementary Fig. 2(b) shows the same analysis with the resonators operated in air, hence in quasi-absence of PEC etching. The mean value is this time zero (no etching) and the standard deviation 7 pm. This indicates that our sweep-cycle tuning experiments are affected by an imprecision of 7 pm in the measurement of the resonant wavelength λ_{WGM}. We anticipate this imprecision to be associated to a lack of spectral accuracy as we sweep the laser wavelength over subsequent cycles (we use rapid sweep scans of an external cavity diode laser). While this technical aspect seems to currently limit the precision of our resonant PEC tuning in the sweep-cycle mode, it is absent in the continuous tuning mode. Simple developments should hence allow improving the (already high) precision shown in the main text.

Supplementary Note 2: Resonant cavity-enhanced PEC tuning of three cavities

Supplementary Figures 3 and 4 provide supplementary data on the collective PEC tuning of three detuned GaAs resonators. Supplementary Fig. 3 shows the set of three disk resonators, imaged at different scales, in order to allow visibility of the optical coupling waveguide, and visualizing the immersion in a droplet of liquid water. Supplementary Fig. 4 shows a series of nine optical spectra acquired on a set of three distinct disks placed around the same optical coupling waveguide, in the configuration shown in Supplementary Fig. 3. The out-of-resonance optical transmittance of the waveguide remains constant in each optical spectrum. More generally, the resonant PEC tuning method does not lead to appreciable changes in the optical transmittance of the tuned devices.

Supplementary Note 3: Residual non-selective PEC tuning of cavities

As visible in the data of Fig. 4(a), there is a residual non-selective etching that takes place in our tuning experiments, at least in the sweep-cycle mode. Its amplitude varies from one experimental situation to another. In Fig. 4(a), it happens to be large (about 4 nm). In Supplementary Fig. 4, it is more moderate (less than 1 nm). Here we clarify the origin of this residual non-selective etch and discuss how it can be controlled in future experiments.

We have identified three sources of residual non-selective etching:

Firstly, it is little known but GaAs gets (very) slowly etched in water. This means that even in the absence of light, a GaAs photonic resonator immersed in water changes dimensions and experiences a slow drift of its resonances. The very small amount of matter dissolved makes it hardly measurable, but an etch speed of 36 pm.min^{-1} was reported [1]. We measured the shift of our GaAs disks WGM resonances in water without light, during several tens of minutes, and deduced an etch speed of 42 ±8 pm.min^{-1}, which for a 1 μm radius disk is also the amount of WGM wavelength shifting. This value is consistent

with the extrapolation at zero power of the power-dependent data shown in Fig. 2(d) of the main text. In consequence, in order to limit the amount of non-specific etching in the liquid, one shall first limit the duration of PEC tuning operations. In the present report, this aspect is not optimized at all; hence, some residual non-selective etching is taking place in water. In humid air, the phenomenon is reduced a lot (we estimated a difference of about six orders of magnitude in the etching speed between water and ambient humid air, see main text).

Secondly, even when laser light is resonantly injected into the WGM of a first disk (Disk 1), while detuned from the WGM resonance of another disk (Disk 2), there is still some optical power injected into that second disk. This undesired power decreases when the Q of resonances increases, or when the detuning between both WGM resonances increases, but it must be accounted for. In Supplementary Fig. 5, we have calculated the ratio between the powers circulating in Disk 1, Disk 2 and in their common coupling waveguide, in a configuration where the disk/guide evanescent coupling conditions are equal for both disks, with a contrast of the WGM resonance of 0.7 and a loaded Q of 22,000 (black curve) or 220,000 (blue curve). The laser is exactly resonant with the WGM of Disk 1 and detuned from that of Disk 2 by a wavelength detuning $\Delta\lambda$. For the loaded Q of 22,000, this configuration is basically that of the experiments reported in Fig. 4(a) of the main text. The case with loaded Q of 220,000 illustrates the trend when Q increases. The calculations are done for a varying detuning, and are based on the standard coupled-mode-theory treatment of disk/guide configurations. For a detuning $\Delta\lambda$=1nm, the circulating power in Disk 2 is 10^{-3} of that in Disk1 (for a Q of 22,000), implying a difference of the same factor in the PEC etching rates. Hence a disk-to-disk PEC tuning selectivity of 10^3 is expected at a spectral distance of 1 nm in the experiments of Fig. 4(a). In the collective PEC tuning, this selectivity progressively drops when the two disks merge spectrally, until they are perfectly aligned.

Thirdly, even when the laser is largely detuned from any WGM resonance, such that zero power is injected into any disk, there is still light travelling in the waveguide. Hence in liquid a (non-resonant) PEC etching occurs at the guide's interfaces, reducing its dimensions. Because the guide sits in the evanescent part of the WGM modes and dispersively acts on them, the WGM resonances are blue-shifted by this etching. Electromagnetic numerical simulations can evaluate this effect. In the experimental conditions of Fig. 4(a), they show that etching 1 nm in the lateral dimensions of the waveguide produces a blue shift of the WGM wavelength of 5 pm. In comparison, etching 1 nm in the radius of the disk produces a WGM shift of 1 nm. Relatively, the effect of waveguide etching is 200 times smaller. We carried out supplementary in-liquid experiments with 20 µW of optical power in the waveguide, during few tens of minutes and in a largely detuned configuration, and measured a WGM wavelength shifting of 75 pm.min^{-1}. Knowing the contribution due to water etching without light, we deduce 33 ±8 pm.min^{-1} of WGM shifting due to non-selective waveguide etching, at this specific optical power. This is commensurable with the amount of water-induced etching without light. At the smaller optical power generally employed in our tuning experiments, this waveguide contribution is however greatly reduced.

With all these elements at hand, we can establish a hierarchy in the mechanisms of non-selective PEC tuning observed in Fig. 4(a), in the sweep-cycle tuning mode. At $\Delta\lambda$=1 nm, the power circulating in the waveguide is 5 times larger that in Disk 2 (see Fig. S5), but since the waveguide etching has relatively 200 times less influence on WGM resonance shifting, the tuning due to waveguide etching is finally 40 times smaller than that due to etching of Disk 2. For the same $\Delta\lambda$=1 nm, the tuning due to etching of Disk 2 is a factor 1000 smaller than the selective tuning of Disk 1. However finally in Fig. 4(a), the measured selectivity is far from such a factor 1000, which indicates that the slow etching of GaAs in water (without light) must be responsible for most of the non-specific tuning. In Fig. 4(a), the sweep-cycle tuning mode was employed and no specific attention was paid to reduce the time interval between subsequent tuning cycles and acquisitions of spectra, generating unnecessary exposure to water and a sizable non-specific etching.

We further support this analysis by looking at another set of data taken in similar conditions, but on two resonators instead of five. In Supplementary Fig. 6, we control more precisely the duration of each tuning step (2 min per step). The power in the waveguide is 2 µW. According to our previous analysis, at this power WGM shifting due to waveguide etching becomes 3.3 ±1 pm.min^{-1}, hence a blue shift of 6.6 ±2 pm per 2-minute cycle. Concomitantly, the water etching without light produces a blue shift of 84 ±16 in 2 minutes, which is more than 10 times larger. Over the total of 6 cycles shown below, these two mechanisms produce overall a non-selective shift of 504 ±96 pm, which is indeed what is observed in Supplementary Fig. 6.

In conclusion, in our current in-water PEC tuning experiments in the sweep-cycle tuning mode, the slow etching of GaAs in water in the absence of light is dominantly responsible for the non-specific tuning, and has currently limited our selectivity to a value of about 100 at best for conditions similar to those of Fig. 4(a). Using the continuous-tuning mode instead of sweep-cycles, operating in a gas atmosphere of controlled humidity, and using larger Qs, will all allow mitigating this non-specific tuning and further increase the selectivity.

Supplementary References

1. Sioncke, S. D. et al. Etch rates of Ge, GaAs and InGaAs in acids, bases and peroxide based mixtures. ECS Transaction 16, 451- 460 (2008).
